# Disease spectrum and medical complaints among migrants: a study from three outpatient clinics for asylum seekers in Berlin, Germany

**DOI:** 10.3389/fpubh.2026.1774309

**Published:** 2026-06-12

**Authors:** Juliane Seidel, Sabine Vygen-Bonnet, Navina Sarma, Joachim Seybold

**Affiliations:** 1 Robert Koch Institute, Department for Infectious Disease Epidemiology, Unit for Crisis Management, Outbreak Investigations and Training Programs, Focal Point for the Public Health Service, Berlin, Germany; 2 Robert Koch Institute, Department for Infectious Disease Epidemiology, Immunization Unit, Berlin, Germany; 3 Charité – Universitätsmedizin Berlin, Medical Directorate, Berlin, Germany

**Keywords:** access to primary care, Outpatient clinics, Primary Health Care, Refugees, Signs and Symptoms, Transients and Migrants

## Abstract

**Introduction:**

In 2015, over 20 million people were compelled to flee due to armed conflict and natural disasters. More than one million arrived in Germany, around 55,000 in Berlin. This study examines health issues among asylum seekers and refugees (ASR) in three newly established outpatient clinics in Berlin's refugee shelters.

**Methods:**

In a cross-sectional study, patient data recorded between 10 September 2015 and 9 March 2016 were extracted retrospectively, entered into an anonymized database, and analyzed. The International Classification of Diseases (ICD10) was used to code diagnoses retrospectively. Stata15/17 was used for data analysis.

**Results:**

A total of 11,894 consultations were included. Of these, 34% (3,774 out of 11,233 consultations for which patient age data was available) were children under five (1,998). Most adults (64%, 4,786/7,459) were aged 21–40. Information on patients' sex was available for 10,898 consultations, 60% (6,556/10,898) were male. Fifty-two countries of origin were represented, Syria (42.6%, 4,263/10,898) and Afghanistan (22.6 %, 2,260/10,898) were the most common. A total of 13,902 diagnoses were documented, 8,818 (67.1%, 8,818/13,135, age-specific data available) were assigned to adults. Infectious diseases made up 46.8% (6,502) of all diagnoses, while 53.2% (7,400) were non-communicable. They were the main diagnoses among children (72%, 3,129/4,317 age-specific data available). Most common for adults and children were respiratory tract infections (75.7%, 4,987). Digestive and circulatory diseases were most frequent in adults (575, 24.1% and 444, 18.6%, respectively). Children suffered most of dermatological conditions and digestive disorders (23.5%, 135/578 and 23%, 132/578, respectively). Orthopedic conditions were the third most common (7.05%, 980/13,902), affecting more adults (9.3%, 820/8,818) and more males.

**Conclusion:**

This study shows a high demand for easily accessible primary healthcare for ASR, upon arrival and during stay in shared accommodation. Most reported diseases corresponded to a ubiquitous general medical spectrum and could be treated in outpatient clinics. The broad range of diseases and complaints demonstrates the interdisciplinary nature of refugee healthcare. Although this study showed a snapshot of the situation, study population size and the consistency of the findings with other studies suggest that the data can be applied to similar situations or settings.

## Introduction

People are forced to flee their countries due to war, conflict, human rights violations, the effects of disasters, climate change and economic hardship. In 2015, the United Nations High Commissioner for Refugees (UNHCR) reported a global increase in the number of displaced persons, reaching a record-high of 65.3 million individuals (1). The number of internally displaced persons, asylum seekers and refugees (ASR) worldwide was 40.8 million, 21.3 million and 3.2 million respectively (1). A significant number of these people originate from Syria (4.9 million), Afghanistan (2.7 million), and Somalia (1.1 million) (1). The majority sought refuge primarily in neighboring countries, with a smaller proportion fleeing to Europe. Germany has become a destination country for people seeking international protection to varying degrees. Around 5.6 million people have applied for asylum in Germany between 1990 and 2022 (2). Large numbers of ASR reached Europe in summer 2015 and more than one million arrived in Germany (2–4) with the highest numbers at the end of 2015 until summer 2016 (see supplementary annex Figure 1, (5)). According to the Federal Office for Migration and Refugees, 441,899 first registration applications were submitted in Germany in 2015 and 722,370 in 2016. The main countries of origin were Syria, Afghanistan, Iraq, Iran, and Eritrea (5–7).

**Figure 1:**
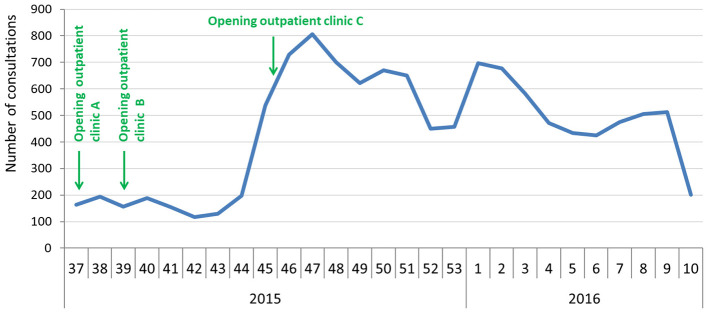
Number of consultations per week at three refugee outpatient clinics in Berlin, 2015/2016 (*n* = 11,894).

In 2015, Berlin (approx. 3.5 million inhabitants) received 55,001 asylum seekers, mostly coming from Syria, Afghanistan, Iraq, and the Republic of Moldova^1^. In 2016, another 16,889 people arrived in Berlin. (8, 9) Berlin's reception facilities and long-term accommodation centers, with a combined capacity of 15,000 places, were already at full occupancy before arrivals increased in summer 2015. The authorities opened large makeshift emergency shelters in gyms, exhibition halls, former airport terminals, and empty buildings, housing around 28,800 ASR (10).

The state of health of many ASR is impaired by war and the strenuous or traumatic experiences of displacement and flight (3, 7). In accordance with Section 62 of the Asylum Act (AsylG), all arriving asylum seekers receive a mandatory physical examination for communicable diseases in order to prevent transmission in shared accommodation centers and, if necessary, to initiate specific treatment (11). In addition the Asylum-Seekers Benefits' Act (12) entitles ASR to limited medical services within the regular healthcare system (basic medical care in case of emergencies, acute illnesses, and pain conditions). Due to a significant delay of many weeks in the initial registration process, access to medical care for unregistered ASR was difficult. Further obstacles included the absence of multilingual services and the limited institutional preparedness within the German healthcare system. Additionally, the existing healthcare structures in many specialist areas (e.g. pediatric, psychiatric capacities) were already operating at the limit of their capacity (4, 7). In order to provide a fast response to the situation, Charité–Universitätsmedizin Berlin established temporary outpatient clinics in the two newly established largest emergency shelters and at a new registration facility for ASR. The primary objective of the initiative was to ensure the provision of fundamental healthcare services, easily accessible and free of charge on-site (7, 13).

In this paper, we describe the demographic characteristics and disease spectrum of ASR who have attended these three Charité outpatient clinics in Berlin between September 10, 2015 and March 9, 2016. During those six months around 43,400 ASR arrived in Berlin (8). This analysis of a large dataset of ASR who have consulted easily accessible outpatient clinics aims to contribute information to support the further planning of medical care for ASR in emergency shelters and to ensure immediate, low-threshold and high-quality care for those without access to the regular healthcare system. The data underlying this study was collected approximately 10 years ago. We are convinced that its significance remains as relevant today as it was then. Therefore, we decided to analyse this previously unexplored data set and publish the results.

## Methods

### Setting, study period and data collection

During summer 2015, 95 emergency refugee shelters were successively opened in Berlin, housing between 500 and 2,500 ASR each (for 28,785 in total) (10). The setup of those shelters varied according to the site and its former purpose (14). The living conditions were characterised by overcrowding, poor hygiene and sanitation, a difficult psychosocial situation, and mass catering services.

Charité university hospital established two temporary outpatient clinics (A and B) on site in two mass accommodation centers (which housed 1,000 and 1,700 people respectively). Those were the largest emergency shelters at the time in Berlin. Outpatient clinic A in former military barracks was operated from September 10 to November 9, 2015. Outpatient clinic B in a large gym was in operation from September 22, 2015 to March 30, 2016. A third outpatient clinic (C) was set up on-site at the Berlin refugee registration office in an administration building from November 2, 2015 to March 30, 2016.

Charité personnel (specialists of various disciplines, non-specialist physicians and midwives) attended patients with the organisational assistance of paramedical staff or volunteers five to six days a week (7). The three outpatient clinics used simply furnished rooms with basic technical means, placed in or in direct proximity to the dormitories or the registration offices, respectively. Basic drugs were provided. Patients could be transferred to hospital by ambulance services if needed. At this time, the three outpatient clinics did not undertake any screening activities as the ASR were highly mobile and were not available for possible follow-up visits. The mandatory x-ray-based TB screening for adult men and non-pregnant women was carried out elsewhere though rather sporadic at this time. The conduction of the statutory medical entry examination addressing all arriving ASRs was set up only in March 2016 in a separate center. Vaccinations based on the recommendations of the German Standing Committee on Vaccination were offered in through campaigns in the screening center and at the accommodations.

Physicians and midwives documented each consultation on a paper form. For each follow-up visit, a separate form was used. There was no ID assigned to the patients, so the forms could not be matched. The form included sociodemographic data (e.g. name, date of birth or age, sex, nationality, language, date of consultation, follow-up consultation) and medical data (e.g. symptoms, medical history, pain scale, vital signs, body sizes, on site diagnostic results, e.g. electrocardiogram, results of rapid urine test, rapid test for StrepA and RSV, diagnosis, therapy/medication, hospitalisation) (details see Supplementary annex table 1). Due to limited diagnostic equipment in the setting, diagnoses were mostly syndromic. Given the limited IT infrastructure, an electronic patient file could not be established. We counted a consultation as a follow-up visit if the “follow-up consultation” field on the form was completed.

### Study design and population

A cross-sectional study was conducted in which patient data recorded by attending physicians or midwives between 10 September 2015 and 9 March 2016 was retrospectively analyzed in 2025. Regarding seasonality, the three outpatient clinics operated from early September to early March. While this period covers different seasonal phases (autumn and winter), all included outpatient clinics operated more or less simultaneously during this time. Therefore, any seasonal factors affecting the disease patterns (e.g., the winter peak in respiratory infections) had a similar impact on all outpatient clinics and ASR treated there. The data were obtained from consultations at easily accessible outpatient clinics, which patients attended primarily due to acute medical complaints. All three outpatient clinics included in this study were identically equipped and operated under very similar conditions.

Documents were excluded if they were illegible due to poor handwriting. The study protocol was reviewed and approved by the Clinical Ethics Committee of Charité–Universitätsmedizin Berlin and the data protection officer of the Robert Koch Institute. The study population included all ASR in Berlin who sought medical care at any of the three Charité outpatient clinics and for whom written documentation was available. Demographic variables such as country of origin or age were documented as stated by the patients and not verified. Data from unaccompanied minors is not included as they were accommodated in designated housing elsewhere.

Due to the available data, individuals cannot be clearly identified. Therefore, our findings refer to consultations or diagnoses and not to incidence/prevalence at the individual level. The individual outpatient clinics did not keep a register of patients treated. It is likely that patients used the outpatient clinic in their shelters as well as in the registration center. Furthermore, some consultations were probably follow-up visits (e.g. for prescriptions). This was not consistently documented. Thus, a certain overlap of populations was expected.

### Definitions

For the purposes of analysis, the patients were categorised according to age groups: 0 to 10 years, 11 to 20 years, 21 to 30 years, 31 to 40 years, 41 to 50 years, 51 to 60 years and over 60 years. Furthermore, adults were categorized into two groups: young-middle-aged (aged 21 to 40) and older adults (aged 41 to >60). Unless otherwise specified and for the sake of brevity, the term “children” refers to individuals below the age of 18 years.

Infectious or communicable diseases were defined according to the WHO-definition: Diseases caused by pathogenic microorganisms (bacteria, viruses, parasites or fungi) with potential of transmission, directly or indirectly, from one person to another; transmitted through bites from insects, or are caused by ingesting contaminated food or water (15). Our definition for non-communicable diseases was: chronic or acute medical conditions which are not infectious and not transmissible from one person to another.

### Data entry, coding, and analysis

Data were manually extracted from the structured documentation forms and entered into a database in an anonymous manner via an EpiData Entry^©^ (16) mask. Two student assistants were trained for data entry and diagnosis category allocation and provided with a standardised data entry manual. A unique identification number was allocated to each form. All diagnoses were entered in their original wording and assigned to syndromic diagnosis categories. For better comparability with other studies, diagnoses were retrospectively coded according to the International Classification of Diseases (ICD10). Depending on how precisely a diagnosis was documented, a specific ICD10-code (e.g. “K59.0” constipation) or a broader ICD10-category (e.g. “K20-K31” diseases of esophagus, stomach, and duodenum) was assigned. We have divided some frequently named diagnoses from the standard ICD10 groups to give a more detailed description, e.g. ICD10 group “M40-M54” (dorsopathies) was modified to “M40-M53” (dorsopathies, excl. back pain/ lumbago) and “M54,-” (Dorsalgia).

Since ICD10 codes are organ-specific, we have created additional categories according to medical specialties. We have also divided the internal medicine category into infectious and non-communicable diseases. Accordingly, seven disease categories were defined:

1. Infectious diseases
2. Non-communicable diseases not belonging to categories 3 to 6
3. Orthopedic conditions: diseases of non-infectious origin related to trauma and disorders of the musculoskeletal system
4. Gynecology (non-infectious) and obstetrics
5. Psychological and behavioral disorders
6. Dental problems: all dental and oral cavity complaints
7. Others

To conduct a more in-depth analysis of these disease categories, subcategories for frequent conditions were created. For example, the subcategory “respiratory infections” included “acute upper respiratory infection” (ICD10 “J06.9”) and “acute bronchitis” (ICD10 “J20.09”) as well as corresponding symptom-related codes, e.g. cough (“R05”).

In this explorative study we described all diagnoses overall and by age, sex and country of origin using frequency and proportions. We calculated median age and interquartile range (IQR). For differences in categorical variables we used Chi^2^-test. We estimated adjusted odds ratio (aOR) and their 95% confidence interval (CI) using logistic regression for each diagnosis by place of origin adjusted for age and sex. We conducted logistic regression in age groups and adult-child status respectively (details see Supplementary annex Table 2). Analyses were performed using Stata15 and Stata17 (17). Data for using an external reference group (such as the German general population) for the calculation of Odds Ratios (OR) were not available. Thus, we used ASR from Syria as an internal reference group. The rational for selecting ASR from Syria was that they were the largest and most stable population group, with a comparable exposure risk to health hazards as other groups. No further adjustments were made regarding the type of outpatient clinic or specific weeks, as all outpatient clinics were equipped identically and the operation of the three outpatient clinics (September to March) differed little from one another, allowing for comparable environmental and seasonal conditions. Due to the lower frequency per country, patients originating from Africa were grouped by region (North Africa, East Africa, West Africa, Central Africa) to avoid very small subgroups. A *P*-value of < 0.05 was considered statistically significant.

## Results

### Demographic characteristics and consultations

We collected in total 12,107 documentation forms of patient consultations from all three locations. We excluded 199 forms due to illegibility. Of the 11,894 included consultations, 6,349 (53.4%) were held at outpatient clinic C, 4,277 (36.0%) at outpatient clinic B, and 1,268 (10.7%) at outpatient clinic A. The frequency of documented consultations ranged from 117 to 807 weekly (see Figure 1). Documented reassessment or follow-up consultations amounted to 3.4% (*n* = 407/11,894) of the consultations.

Details on patient age were available for 11,233 consultations. The mean age was 24 years (interquartile range (IQR): 9–35). Of these 7,459 (66%) were adults and 3,774 (34%) were children. Of the children, 1,998 (18% of all consultations) were under 5 years of age. Most adults (64%, 4,786/7,459) and 43% of all patients (4,786/11,233) were between 21 and 40 years old (see Supplementary annex Table 3 “Age of patients in age groups”).

**Figure 2:**
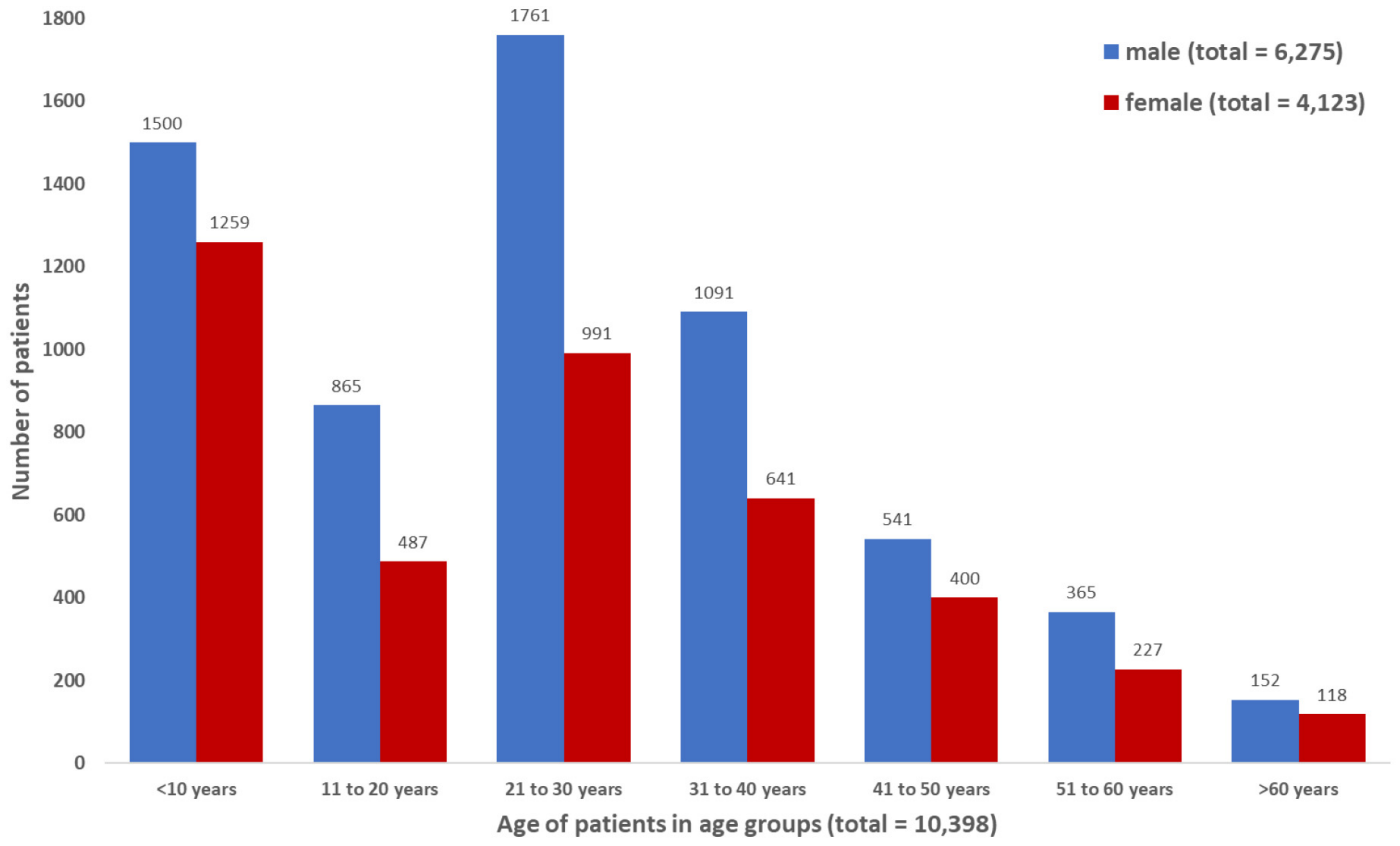
Age and sex distribution of ASR consulting three refugee outpatient clinics in Berlin, 2015/2016 (*n* = 10,398, data on patient age and sex available).

The services in outpatient clinics A and C were predominantly (75%) used by adults (814/1,089 and 4,613/6,143, respectively; *p* < 0.001), while at outpatient clinic B children and adults were equally represented. Information on patients' sex was available for 10,898 consultations, 60% (6,556/10,898) were male and 40% (4,342/10,898) female. Datasets with age and sex were available for 10,398 consultations (see Figure 2).

Information about the patient's country of origin was available for 84% (10,019/11,894) of all consultations. The attending patients came from 52 different countries, with Syria (42.6%, 4,263) and Afghanistan (22.6%, 2,260) being the most common (see Table 1 and Supplementary annex Table 4). Most frequent regions of origin were “South-western Asia and Middle East” (65.5%, 6,558, including Syria, Iraq, Iran), “Southeast and South Asia” (24.7%, 2,473, including Afghanistan, Pakistan) followed by the “Commonwealth of Independent States (CIS)” and the “Balkans” (7.3%, 727), and Africa (2.6%, 261).

**Table 1:** Countries of origin of refugees consulting (grouped, available information on country of origin for *n* = 10,019 consultations) at all three outpatient clinics in Berlin, 2015/2016.

Country of origin	Number	Percent
Syria	4,263	42.6
Afghanistan	2,260	22.6
Iraq	1,575	15.7
Iran	489	4.9
CIS (former Soviet republics in Eurasia; Armenia, Belarus, Chechnya, Georgia, Kazakhstan, Latvia, Russia, Turkmenistan, Ukraine, Uzbekistan)	433	4.3
Balkans (Albania, Bosnia, Bulgaria, Kosovo, Macedonia, Romania, Serbia)	294	2.9
Pakistan	197	2.0
North Africa (Algeria, Egypt, Libya, Morocco, Sudan, Tunisia)	151	1.5
East Africa (Kenya, Somalia, Ethiopia, Eritrea, Djibouti, Uganda)	81	0.8
West Africa (Ghana, Guinea–Bissau, Nigeria, Senegal, The Gambia)	24	0.2
Central Africa (Cameroon, Equatorial Guinea)	5	0.1
Other (Bangladesh, Kurdistan, Lebanon, Nepal, Palestine, Turkey, Vietnam, Yemen)	247	2.5
**Total**	**10,019**	**100**

Details on country of origin and age were available for 9,616 consultations. The highest proportion of children was among patients from Afghanistan (44%, 947/2,150) and Syria (33%, 1,362/4,086), (*p* < 0.001). The proportion of Afghanistan as country of origin among children (30%, 947) was higher than among adults (19%, 1,203, *p* < 0.001).

Attending physicians documented in total 13,902 diagnoses; 11,894 (85.6%) consultations with one diagnosis, 2,011 consultations with two to five diagnoses (see Table 2). A total of 13,902 ICD-10 codes were assigned. Of the diagnoses for which age-specific data was available, 67.1% (8,818/13,135) diagnoses were assigned to adults and 32.9% (4,317/13,135) to children. Children under five years of age accounted for 54.6% (2,358/4,317) of patients aged < 18 years.

**Table 2:** Number of diagnoses per consultation (*n* = 13,902, one until up to five diagnosis assigned per patient) at three refugee outpatient clinics in Berlin 2015/2016.

Number of diagnoses per consultation	Consultations (Number)	Consultations (Percent)
1 diagnosis	11,894	85.5
2 diagnoses	1,774	12.7
3 diagnoses	202	1.4
4 diagnoses	29	0.2
5 diagnoses	3	0.02
**Total**	**13,902**	**100**

Referrals to specialists were documented in 17.5% (2,076/11,894) of the consultations (see Table 3). Most referrals (91.4%, 1,904/2,076) were to a single specialist, 7.8% (162/2,076) to two and 17 to three different specialists. 22.8% (473/2,076) of all referrals were for consultation with a general practitioner; 421 of these were from adults (421 of 1,639 referrals among adults). Children were mainly referred to pediatrics (180 of 341 referrals among children). Hospitalisation was documented for 2.8% (338/11,894) of the consultations. 55% of hospitalised patients were male (176/320) and 61.5% were adults (195/317). The distribution of countries of origin among patients who were hospitalised was consistent with the overall distribution. Most common causes for hospitalisation were diseases of the circulatory (ICD10 IX; 11%, *n* = 34) and respiratory system (ICD10 X; 16%, *n* = 21) in adults (*n* = 195), and diseases of the respiratory system (ICD10 X; 23%, *n* = 30) and certain infectious and parasitic diseases (ICD10 I; 18%, *n* = 22) in children (*n* = 122).

**Table 3:** List of specialist's referrals in total (*n* = 2,076 consultations) and by age (*n* = 1,980 consultations with data on age available), one until up to three referrals assigned per patient) among refugees consulting at three outpatient clinics in Berlin, 2015/2016.

Specialist's referrals	≥18 years		<18 years		Total all^*^	
	*n*	%	*n*	%	*n*	%
General medicine	421	25.7	33	9.4	473	22.7
Orthopedics	223	13.6	7	2.0	236	11.4
Gynecology, midwifery/obstetrics	195	11.9	1	0.3	203	9.8
Pediatrics	0	0.0	180	52.8	188	9.0
Dentistry	75	4.6	23	6.7	104	5.0
Ear, nose & throat medicine (ENT medicine)	69	4.2	11	3.2	85	4.1
Dermatology	67	4.1	15	4.4	89	4.3
Psychology, psychiatric clinic	73	4.4	2	0.6	79	3.8
Surgery	70	4.3	8	2.3	83	4.0
Neurology	63	3.8	8	2.3	76	3.7
Accident & emergency department (A&E)	60	3.7	15	4.4	77	3.6
Internal medicine/	67	4.1	1	0.3	70	3.4
Hospital	51	3.1	14	4.4	69	3.3
Ophthalmology	48	2.9	7	2.0	59	2.8
Cardiology	45	2.7	3	0.9	52	2.5
Urology	44	2.7	4	1.2	52	2.5
Function diagnosis/diagnostics	16	0.9	2	0.6	19	0.9
Pneumology	18	1.1	2	0.6	21	1.0
Oncology	13	0.8	1	0.3	15	0.7
Other	21	1.3	4	0.3	27	1.3
**Total**	**1,639**	**100**	**341**	**100**	**2,076**	**100**

^*^The total comprises of the sum of the two first columns plus the consultations for which age was not indicated.

**Table 4:** List of ICD10 codes of subcategory “Infectious diseases” by age (*n* = 6,141, data on age available) and in total (*n* = 6,502); patient data from three refugee outpatient clinics in Berlin, 2015/2016.

Subcategory–infectious diseases		≥18 years		<18 years		Total all^*^	
		*n*	%	*n*	%	*n*	%
**Respiratory infections (incl. TB, bronchitis, pneumonia) & Otitis**		**2,334**	**77.5**	**2,377**	**76**	**4,987**	**76.7**
J06.9	Acute upper respiratory infection, unspecified	1,983	65.8	2,028	64.8	4,254	65.4
J20.09	Acute bronchitis, unspecified	197	6.4	203	6.5	418	6.4
H60, H66–H68	Otitis externa/media, Eustachian salpingitis and obstruction	64	2.1	105	3.4	177	2.7
J18.–	Pneumonia, organism unspecified	53	1.7	39	1.2	96	1.5
A15–A19	Tuberculosis	37	1.2	2	0.1	42	0.6
**Helminthiases/Pediculosis**		**140**	**4.6**	**122**	**3.9**	**282**	**4.3**
B85–B89	Pediculosis, ascariasis and other infestations	137	4.4	113	3.5	270	4.1
B65–B83	Helminthiases	3	0.1	9	0.3	12	0.2
**Gastrointestinal infections**		**135**	**4.4**	**423**	**13.5**	**586**	**8.9**
A09 (Other gastroenteritis, colitis of infectious/unspecified origin)							
**Urinary tract infections**		**131**	**4.3**	**23**	**0.7**	**162**	**2.5**
N39.0	Urinary tract infection, site not specified	123	4.0	20	0.6	150	2.3
N30.9	Cystitis, unspecified	8	0.3	3	0.1	12	0.2
**Skin infections (incl. viral infections characterized by skin lesions)**		**126**	**4.0**	**102**	**3.2**	**243**	**3.6**
L00–L08	Infections of the skin and subcutaneous tissue	101	3.6	42	1.4	154	2.4
B00–B09	Viral infections characterized by skin, mucous membrane lesions	25	0.3	60	1.8	89	1.2
**Mycoses (B35–B49)**		**72**	**2.4**	**38**	**1.2**	**122**	**1.9**
**Viral infections (other than respiratory and gastrointestinal)**		**51**	**1.7**	**8**	**0.3**	**61**	**0.9**
B25–B34	Other viral diseases	3	0.1	3	0.1	7	0.1
B15–B19	Viral hepatitis	32	1.0	5	0.2	38	0.6
B20–B24	Human immunodeficiency virus [HIV] disease	14	0.5	–	–	14	0.2
A80–A89	Viral infections of the central nervous system	2	0.1	–	–	2	0.03
**Other**		**23**	**0.8**	**36**	**1.15**	**59**	**0.9**
H10.0	Mucopurulent conjunctivitis	10	0.4	18	0.5	28	0.4
A30–A49	Other bacterial diseases	–	–	11	0.3	11	0.2
A50–A64	Infections with predominantly sexual mode of transmission	8	0.3	–	–	8	0.1
G00–G09	Inflammatory diseases of the central nervous system	2	0.1	5	0.2	7	0.1
B50–B64	Protozoal diseases	–	–	2	0.1	2	0.03
B99–B99	Other infectious diseases	2	0.1	–	–	2	0.03
A28.–	Zoonotic bacterial disease, unspecified	1	0.03	–	–	1	0.02
**Total**		**3,012**	**100**	**3,129**	**100**	**6,502**	**100**

^*^The total comprises of the sum of the two first columns plus the diagnoses for which age was not indicated.

### Diagnoses and disease groups

**Figure 3:**
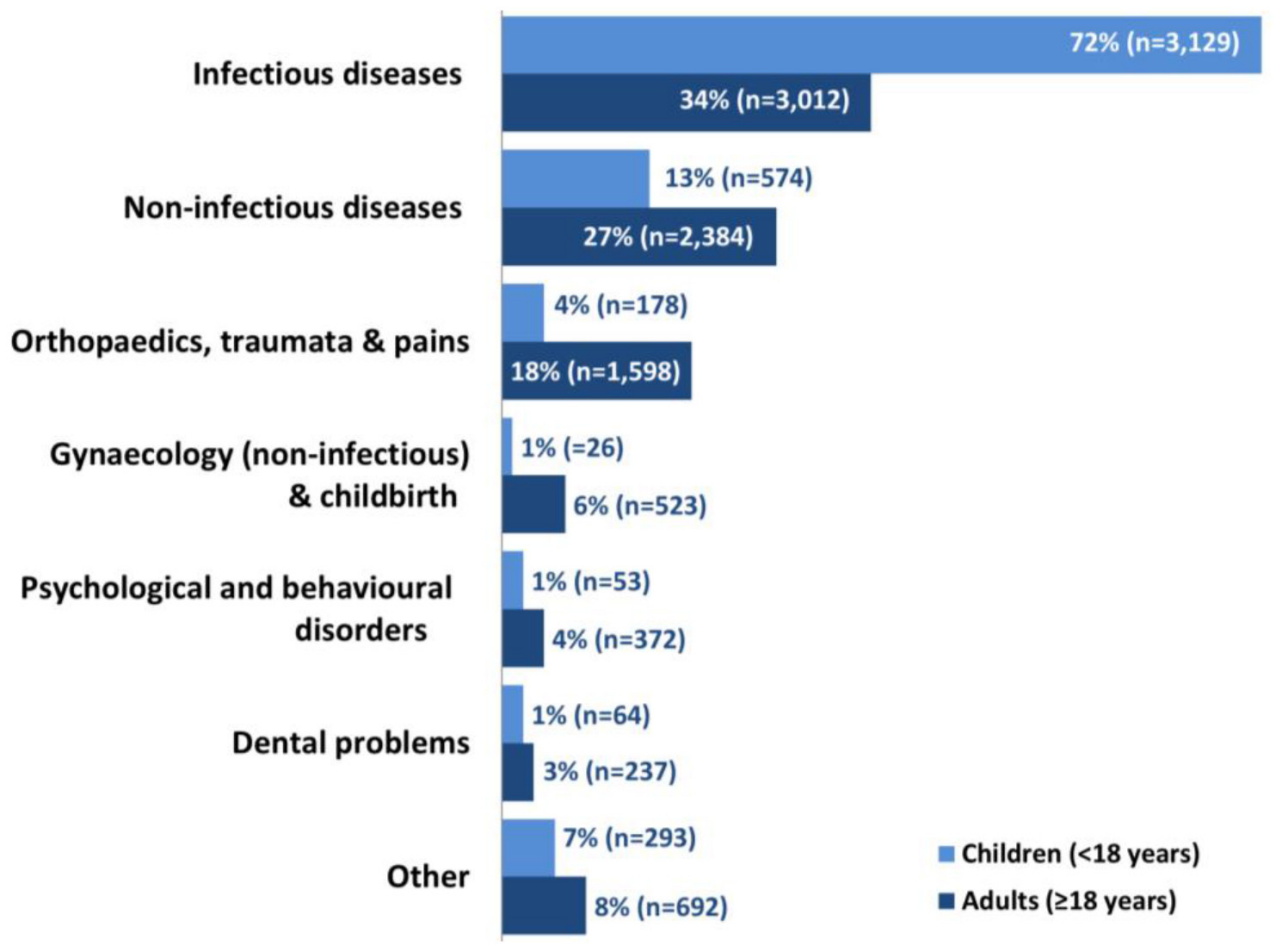
Distribution of diagnoses according to disease categories* by age groups at three refugee outpatient clinics in Berlin, 2015/2016 (*n*=13,135 diagnoses with documented age). * The category “non-infectious diseases” includes all non-communicable diseases which are not included in any other category.

Of a total of 13,902 diagnoses, 6,502 (46.8%) were infectious and 7,400 (53.2%) were non-communicable diseases including gynecology, obstetrics, orthopedics, etc. Of all conditions, 67.1% (8,818/13,135 diagnoses with age-specific data available) were diagnosed in adults. Infectious diseases were the main diagnoses in children (see Figure 3 and Supplementary annex Table 5).

### Subgroup analyses

#### Infectious diseases

Overall, children were more affected by infectious diseases than adults (72.5%, 3,129/4,317 versus, 34.2%, 3,012/8,818, *p* < 0.001), particularly children up to 10 years of age (76%, 2,696/3,549, *p* < 0.001). The most prevalent infectious diseases in both consultations from adults and pediatric were respiratory tract infections (RTI; 75.7%, *n* = 4,987) (see Table 1).

Adjusted for sex and age group, consultations from patients from Afghanistan and Iran had a significantly higher odds for infectious disease [aOR 1.42 (95% CI: 1.27–1.58) and 1.39 (95% CI: 1.15–1.68), respectively], and consultations from patients from the CIS, the Balkans, and Africa a lower odds [aOR: 0.80 (95% CI: 0.64–0.99), aOR 0.49 (95% CI: 0.37–0.65) and aOR 0.46 (95% CI: 0.34–0.62), respectively].

Most infectious diseases in children affected the respiratory tract (76%; 2,377/3,129). RTI made up 55% (2,377/4,317) of all diagnosis in children versus 26.5% (2,334/8,818; *p* < 0.001) in adults [aOR 3.47 (95% CI: 3.18–3.79)]. Particularly affected were children under five years of age (58.4%, 1,377/2,358) compared to 30.9% among all other ages (3,334/10,781; *p* < 0.001). Most affected were patients from Afghanistan (45.1%, 1,172/2,598; *p* < 0.001). Among all 4,987 RTI were 88.9% (*n* = 4,431) lower respiratory tract infections (LRTI) and 11.2% (*n* = 556) upper respiratory tract infections (incl. otitis, URTI). 1.3% of RTI needed hospitalisation (*n* = 65/4,987, 14.2% of all 459 hospitalisations; *p* < 0.001), 26 due to LRTI as pneumonia (*n* = 16), bronchitis (*n* = 6) and tuberculosis (*n* = 4), as well as 39 due to URTI (viral and bacterial, incl. tonsillitis). 64.5% of hospitalised patients were under five years old (40/62), mostly hospitalisation for pneumonia, bronchitis (*n* = 15) and URTI (*n* = 18). Less than one percent (42/4,987) of RTI was recorded as “reported tuberculosis”. Among the consultations with diagnosis tuberculosis was one infant. In three consultations the age was not indicated, and all others were adults, mostly (71%; 28/39) aged between 21 and 40. Thirty-six were men and five were women (one patient's sex was not recorded). Fifteen of the TB diagnoses were among ASR from Syria and seven from the CIS.

Gastrointestinal infections (*n* = 586) were the second most frequent infectious diseases, e.g. diarrhea, infectious gastro-enteritis or enteritis (ICD10 “A09”). 76% of all gastrointestinal infections were among children (423/558) and 24% among adults [135/558; *p* < 0.001; aOR 6.3 (95% CI: 5.03–7.90)]. Children under five years of age were more affected than all other age groups [aOR 5.63 (95% CI: 4.60–6.90)].

Of the 282 parasite infections, 95.7% (270/282) concerned the skin. The relative frequency in children was significantly higher than in adults (2.8%, 122/4,321 versus 1.6%, 140/8,818, *p* < 0.001). 89/262 infections occurred in the 0 to 10 age group. Consultations from ASR from Pakistan and from African countries had a significantly higher odds for parasite infections [aOR 5.47 (95% CI: 3.16–9.44) and aOR 2.35 (95% CI: 1.27–4.35), respectively].

Also, skin infections and diseases manifesting on the skin affected more children than adults (2.3%, 101/4,317 versus 1.4%, 122/8,818, *p* < 0.001). Of 243 skin rashes, 18.1% (44/243) were attributable to varicella virus infections, with 83.7% (36/43) of these occurring in the 0 to 10 age group.

Women (113/152) had four folds higher odds of urinary tract infections (UTI, ICD10 N30.9, N39.0, *n* = 162) than men [aOR 4.46 (95% CI: 2.99–6.65)].

#### Non-communicable internal medical diseases

In the domain of internal medical conditions (N = 3,125 with age-specific data available), diseases of the digestive and circulatory systems were the most prevalent in adults, 24.1% (24.1% 575/2,384) and 18.6% (444/2,384), respectively (see Table 5). In contrast, children exhibited the highest prevalence of dermatological conditions, including dermatitis and eczema, as well as digestive system disorders (23.5%, 135/574 and 23%, 132/574, respectively).

**Table 5:** List of ICD10 codes of subcategory “Non–communicable internal medical diseases” by age (*n* = 2,958, data on age available) and in total (*n* = 3,125); patient data from three refugee outpatient clinics in Berlin, 2015/2016.

Superordinate category–Non–communicable internal medical diseases		≥18 years		<18 years		Total all^*^	
		*n*	%	*n*	%	*n*	%
**XI**	**Diseases of the digestive system**	**575**	**24.1**	**132**	**21.3**	**750**	**23.6**
R10–R19	Symptoms and signs involving the digestive system and abdomen	93	3.9	79	12.8	182	5.7
K29.7	Gastritis, unspecified	140	5.9	8	1.3	155	4.9
K59.0	Constipation	85	3.6	18	2.9	110	3.5
K20–K31	Diseases of esophagus, stomach and duodenum	88	3.7	7	1.1	103	3.2
K55–K64	Other diseases of intestines	84	3.5	5	0.8	96	3.0
K40–K46	Hernia	32	1.3	2	0.3	34	1.1
K35–K38	Diseases of appendix	15	0.6	7	1.1	26	0.8
K80–K87	Disorders of gallbladder, biliary tract and pancreas	15	0.6	1	0.03	16	0.5
K65.–	Peritonitis	2	0.1	–	–	2	0.1
K50–K52	Noninfective enteritis and colitis	9	0.4	3	0.1	12	0.4
K70–K77	Diseases of liver	8	0.3	–	–	8	0.3
K90–K93	Other diseases of the digestive system	4	0.2	2	0.32	6	0.2
**IX**	**Diseases of the circulatory system**	**444**	**18.59**	**37**	**5.99**	**501**	**15.8**
I00–I99^**^	Diseases of the circulatory system	197	8.3	35	5.7	238	7.5
I10–I15	Hypertensive diseases	160	6.7	2	0.3	174	5.5
I20–I25	Ischaemic heart diseases	75	3.1	–	–	77	2.4
I60, I62–I69	Cerebrovascular diseases	11	0.5	–	–	11	0.4
I61.–	Intracerebral hemorrhage	1	0.04	–	–	1	0.03
**XII**	**Diseases of the skin and subcutaneous tissue**	**239**	**10.0**	**135**	**21.8**	**415**	**13.1**
L80–L99	Other disorders of the skin and subcutaneous tissue	119	5.0	41	6.6	176	5.5
L40.9	Psoriasis, unspecified	10	0.4	2	0.3	14	0.4
R20–R23	Symptoms and signs involving the skin and subcutaneous tissue	58	2.4	28	4.5	94	3.0
L20–L30	Dermatitis and eczema	49	2.1	63	10.2	127	4.0
L50	Urticaria	3	0.2	1	0.2	4	0.1
**IV**	**Endocrine, nutritional and metabolic diseases**	**233**	**9.8**	**22**	**3.8**	**265**	**8.5**
E10–E14	Diabetes mellitus	180	7.5	3	0.5	189	6.0
E00–E90	Endocrine, nutritional and metabolic diseases	49	2.1	5	0.9	57	1.8
E40–E46	Malnutrition	2	0.1	8	1.4	11	0.4
E50–E64	Other nutritional deficiencies	2	0.1	6	0.5	8	0.3
**X**	**Diseases of the respiratory system**	**173**	**7.2**	**60**	**9.7**	**243**	**7.7**
J40–J47	Chronic lower respiratory diseases	135	5.7	39	6.3	179	5.6
R00–R09	Symptoms and signs involving the circulatory and respiratory systems	17	0.7	4	0.7	25	0.8
J30–J39	Other diseases of upper respiratory tract	12	0.5	13	2.1	25	0.8
J90–J99	Other diseases of the respiratory system	7	0.3	4	0.7	12	0.4
J80–J84	Other respiratory diseases principally affecting the interstitium	2	0.1	-	-	2	0.1
**VI**	**Diseases of the nervous system**	**167**	**7.0**	**47**	**7.6**	**227**	**7.3**
G4—	Epilepsy	46	1.9	26	4.2	**78**	**2.5**
G80–G83	Cerebral palsy and other paralytic syndromes	47	2.0	6	1.0	**55**	**1.8**
G35–G37	Demyelinating diseases of the central nervous system	21	0.9	1	0.2	**23**	**0.7**
R56.–	Convulsions, not elsewhere classified	8	0.3	9	1.5	**19**	**0.6**
G50–G59	Nerve, nerve root and plexus disorders	30	1.3	–	–	**32**	**1.0**
G90–G99	Other disorders of the nervous system	5	0.2	2	0.3	**7**	**0.2**
G20–G26	Extrapyramidal and movement disorders	4	0.2	–	–	**4**	**0.1**
G30–G32	Other degenerative diseases of the nervous system	2	0.1	1	0.2	**3**	**0.1**
G45.–	Transient cerebral ischaemic attacks and related syndromes	1	0.04	1	0.2	**2**	**0.1**
G47.–	Sleep disorders	1	0.04	1	0.2	**2**	**0.1**
G60–G64	Polyneuropathies and other disorders of the peripheral nervous system	2	0.1	–	–	**2**	**0.1**
**VII**	**Diseases of the eye and adnexa**	**126**	**5.3**	**56**	**9.1**	**193**	**6.1**
H00–H59^***^	Diseases of the eye and adnexa	126	5.4	56	**9.7**	**193**	**6.3**
**XIV**	**Diseases of the genitourinary system (excl. gynecological manifestations)**	**164**	**6.9**	**30**	**4.9**	**206**	**6.5**
N00–N39	diseases of renal/urinary system (non–communicable)	112	4.7	11	1.8	129	4.1
N40–N51	Diseases of male genital organs	22	0.9	7	1.1	32	1.0
R30–R39	Symptoms and signs involving the urinary system	28	1.2	12	1.9	43	1.4
N60–N64	Hypertrophy of breast	2	0.1	–	–	2	0.1
**II**	**Neoplasms**	**110**	**4.6**	**5**	**0.8**	**117**	**3.7**
C00–C97	Malignant neoplasms	95	4.2	4	0.8	**100**	**3.2**
D10–D36	Benign neoplasms	11	0.5	1	0.2	**13**	**0.4**
D37–D48	Neoplasms of uncertain or unknown behavior	4	0.2	–	–	**4**	**0.1**
**III**	**Diseases of the blood**	**40**	**1.7**	**22**	**3.7**	**63**	**2.0**
D50–D90	Diseases of the blood and blood–forming organs and certain disorders involving the immune mechanism						
**VIII**	**Diseases of the ear and mastoid process**	**37**	**1.6**	**16**	**2.6**	**54**	**1.7**
H65.–, H69–H75	Diseases of middle ear and mastoid (non–communicable)	9	0.4	2	0.32	12	0.4
H61–H62	Other disorders of external ear (non–communicable)	4	0.2	5	0.8	9	0.3
H90–H95^****^	Other disorders of ear	15	0.6	–	–	15	0.5
H80–H83	Diseases of inner ear	6	0.3	–	–	6	0.2
H92.0	Otalgia	3	0.2	9	1.5	12	0.4
	**Allergies**	**60**	**2.5**	**10**	**1.6**	**73**	**2.3**
T78.4	Allergy, unspecified	24	1.0	2	0.3	**29**	**0.9**
L23–	Allergic contact dermatitis	20	0.8	7	1.1	**27**	**0.9**
J30–	Vasomotor and allergic rhinitis	10	0.4	1	0.2	**11**	**0.4**
H10.1	Acute atopic conjunctivitis	6	0.3	–	–	**6**	**0.2**
	**Other**	**16**	**0.7**	**2**	**0.4**	**17**	**0.5**
R55	Syncope and collapse	14	0.6	2	0.3	**16**	**0.5**
M30–M36	Systemic connective tissue disorders	2	0.1	–	–	**2**	**0.1**
**Total**		**2,384**	**100**	**574**	**100**	**3,125**	**100**

^*^The total comprises of the sum of the two first columns plus the diagnoses for which age was not indicated.

^**^ Modified by author, superordinate ICD10 group “I00-I99” exclusive: “I10-I15”, “I20-I25”, “I60-I69”, “I61.-”, they (“I20-I25”, “I60-I69”, “I61.-”) are separately displayed in the list above.

^***^Modified by author, excl. H10.1, H10.1 separately displayed in the list above.

^****^Modified by author, excl. H92.0, H92.0separately displayed in the list above.

Most consultations with gastrointestinal diagnoses were from young adults (344/706, 48.7%; 344/5,558, 6.2%), but in the age groups “51–60” and “41–50” years gastrointestinal diagnoses had the largest shares of 9% and 7.7%, respectively.

92.3% (444/481) of diagnoses with cardiovascular diseases (CVD) were in adults. CVD represented 5% (444/8,818) of diagnoses in adults and 0.9% (37/4,317) in children (*p* < 0.001). ASR consultations from the Balkans and Africa had higher odds of CVD [aOR 1.96 (95% CI: 1.31–2.94), aOR: 1.78 (95% CI: 1.07–2.96), respectively], who also represented the oldest groups among the adult ASR population. Hypertensive diseases (including ICD10 “I10-I15”) were the most frequent conditions among CVD (34.7%, 174/501) and affected significantly more women than men [aOR 1.80 (95% CI: 1.27–2.55)]. For Ischaemic heart diseases (ICD10 “I20-I25”; *n* = 77) there was no difference for sex. The most affected age group was 51–60–years (28/77; 3.6% of all, 28/783; *p* < 0.001). Most affected were ASR from the Balkans [aOR: 3.29 (95% CI: 1. 49–7.24)].

In the category of non-communicable diseases, skin diseases (415/3,125) were the most frequently reported conditions in children (23.5%, 135/574), e.g. napkin dermatitis (*n* = 41, ICD10 “L22”). In adults skin problems made up 10% (239/2,384) of non-communicable diseases, most frequently reported were “various other disorders of the skin and subcutaneous tissue” like e.g. acne, blisters and skin lesion (*n* = 120; ICD10 “L80-L99”), “rash and other nonspecific skin eruption” (*n* = 57, ICD10 “R20-L23”) and “atopic and nonspecific dermatitis” (*n* = 49; ICD10 “L20-L30”).

Among the endocrine diseases (265/3,125) diabetes mellitus made up 71.3% (189/265). Almost all diabetes diagnoses were in adults (98.3%, 180/183). Overall diabetes made up 2% of all diagnoses in adults (180/8,818). As endocrine diseases generally, adults in the age groups 51–60 years (54/180) and over 60 years (23/180) were most affected (*p* < 0.001).

Non-communicable respiratory conditions (243/3,125) concerned 1.4% (60/4,317) of children and two percent of adults (173/8,818; *p* = 0.012) and more male than female [aOR 1.55 (95% CI: 1.14–2.10)], particularly adults in age group 51–60 years old compared to children had three times higher odds for those conditions [aOR 3.13 (95% CI: 1.93–5.09)]. ASR consultations from Afghanistan [aOR 0.44 (95% CI: 0.27–0.70)] had lower odds of non-communicable respiratory conditions compared to consultations from Syria.

The largest subgroup were chronic lower respiratory diseases (74.6%, 179/241, ICD10 “J40-J47”), mostly asthma (71%; 127/179) and chronic obstructive pulmonary disease (COPD) (21.2%; 38/179) and mostly concerning adults (77.6%; 135/174, 1.5%; 135/8,818) of adults versus 22.4% in children (39/174), 0.9% (39/4,321) of all children; *p* = 0.003). As among non-communicable respiratory conditions in general, patients aged 51–60 years were most concerned by chronic lower respiratory diseases (4.1%, 32/783; *p* < 0.001). Asthma was here the predominant diagnosis among children (36/39).

Neurological diseases (227/3,125) affected 1.9% (167/8,818) of adults and 1.1% (47/4,317) of children (*p* = 0.003). The odds of neurological conditions was almost twice as high in diagnoses from consultations of male ASR as for female [aOR 1.96 (95% CI: 1.40–2.75)]. Adults of age group 31–40 years had twice the odds of presenting those conditions compared to all other age groups [aOR 2.10 (95% CI: 1.32–3.35), *p* = 0.002). Patient consultations from the Balkans, the CIS, Iran and Iraq had higher odds compared to Syrians [aOR 2.91, 95%CI: 1.54–5.49; aOR 2.16 (95% CI: 1.19–3.91), aOR 2.12 (95% CI: 1.21–3.72), aOR 1.75 (95% CI: 1.18–2.61), respectively]. Epilepsy (*n* = 78, ICD10 “G40.-”) was the most important neurological diagnosis, age groups were equally concerned (0.5% of all adults (46/8,818), 0.6% of all children (26/4,321; *p* = 0.559). Consultations from ASR from Iran and the CIS were four and three times more affected than those from Syria [aOR 4.16 (95% CI: 1.87–9.24), aOR 3.0 (95% CI 1.19–7.48), respectively].

117 presentations with history or suspicion of neoplasms (ICD10 “C00-D48”) were documented, almost all concerned adults (95.6%, 110/115) with no difference in sex (female: 51/113, 45.1%; 51/5,254, 1.0% of all females versus males: 62/113, 54.9%; 62/7,491, 0.8% of all men; *p* = 0.397). Mainly malignant neoplasms (100/117, ICD10 “C00-C97”) were documented, most frequently carcinomas of the gastrointestinal tract (*n* = 19), “lymphomas and leukemia” (*n* = 13 and *n* = 6), malignant neoplasm of the breast (*n* = 13), and astrocytoma (*n* = 9). Compared to consultations from ASR from Syria, consultations from ASR from the CIS had three times higher odds of malignant neoplasm [aOR 3.32 (95% CI: 1.84–5.97)].

Overall, 52 anaemias were reported, among those were six thalassaemia and four sickle-cell anaemias (ICD10 “D55-D59”). Thalassaemia was documented in patient consultations from Syria (*n* = 2), Iraq and Afghanistan (one each) and all sickle-cell anemia cases from Syria. All were children and young adults.

Among the non-communicable diseases concerning the ear (54/3,125), hearing loss/deafness or tinnitus (15/54, ICD10 “H90-H95”) were most frequent followed by diseases of the middle ear and mastoid, e.g. perforation/rupture of the tympanic membrane (12/54, ICD10 “H65.-, H69-H75”), otalgia (12/54, ICD10 “H92.0”) and disorders of the external ear (9/54, ICD10 “H61-H62”). Adults and children were equally concerned (0.4%, 36/8,818 of all adults and 0.4%, 16/4,317 of all children; *p* = 0.8).

#### Orthopedics

Orthopedic conditions (980/13,902 all diagnoses, 7.1%) constituted the third most prevalent superordinate category. Adults exhibited higher odds of these conditions (9.3%, 820/8,818 age-specific data available) compared to children (2.6%, 114/4,317; *p* < 0.001). Additionally, males demonstrated higher odds than females [aOR 1.70 (95% CI: 1.44–2.0), data on age and sex available]. See Supplementary annex Table 6 for list of all orthopedic complaints.

Injuries (*n* = 318) were a frequent cause for attending the outpatient clinics. According to age-specific data available, injuries were more documented in consultations from young adults from 21 to 30 years than in consultations from under 20 years [11–20 years: aOR 3.94 (95% CI: 2.35–6.61), 21–30 years: aOR 3.59 (95% CI: 2.23–5.792)]. Injuries were mostly diagnosed in male patients [aOR 2.44 (95% CI: 1.77–3.38)]. Most affected were consultations from ASR from the Balkans [aOR 2.04 (95% CI: 1.13–3.71)]. Most injuries were contusions and distortions of joints, ligaments and muscles of limps (143/318, ICD10 “S40-S99” *n* = 192). Wounds, abrasive wounds and cuts made up 53 diagnoses (ICD10 “T08-T14”).

Disorders of the musculoskeletal system, soft tissue, spine and back (*n* = 309) affected predominantly adults (3.2% [283/8,818] versus 0.5% [20/4,317] of children; *p* < 0.001), particularly in 41–50 and 51–60 age groups [aOR 48.83 (95% CI: 15.18–157.08) and aOR 50.45 (95% CI: 15.42–165.0), respectively, *p* = 0.000]. Major diagnoses in this disease group were related to muscle tensions (*n* = 121), disc prolapse (*n* = 51), rheumatic disease (*n* = 16) and deformity of the spine (*n* = 14).

#### Pains

“Pains” (*n* = 893/13,902 all diagnoses) resulted predominantly from orthopedic conditions (91.1%, 893/980), such as dorsalgia (34.7%, 308/893), and limb pain (27.4%, 209/893), followed by migraine and headache (29.9%, 265/893). According to data with information on patients' age and sex available, the odds of reporting pains were higher in males than female patients [aOR 1.18 (95% CI: 1.0–1.40), *p* = 0.044] and in adults compared to children [aOR 6.82 (95% CI: 5.05–9.21), *p* < 0.001]; particularly in middle and young aged adults [41–50 years: aOR 15.49 (95% CI: 9.63–24.89); 31–40 years: aOR 12.69 (95% CI: 8.01–20.12) and 21–30 years: aOR 12.26 (95% CI: 7.81–19.23), *p* < 0.001]. While dorsalgia (ICD10 “M54.-“) and “migraine and headache” (ICD10 “G43-G44”) were documented in consultations of male and female patients equally (2.4%, 183/7,491 of men versus 2.1%, 111/5254 of women, *p* = 0.222 and 1.9%, 145/7491 of men versus 2.1%, 108/5,254 of women; *p* = 0.633), the odds of reported limb pain (ICD10 “M79.6”) were higher in male patients [aOR 1.80 (95% CI: 1.27–2.55], *p* = 0.001).

#### Gynecology (non-communicable) and obstetrics

Regarding obstetrics and gynecology (595/13,902 all diagnoses), the main reason for consultation was antenatal care (54.29%; *n*=323/595; ICD10 “Z34”), followed by non-inflammatory disorders of the female genital tract (18.99%, 113/595; ICD10 “N80-N98”) such as menstrual problems (*n* = 30), vaginal bleeding (*n* = 25), unspecific abdominal discomfort (*n* = 22), myoma (*n* = 10) and ovarian cysts (*n* = 9). For detailed information see Supplementary annex Table 7.

A total of 447 pregnancies (5,254 consultations with women) were documented, with the age also specified in 414 pregnancies (4,995 consultations with women). Of these, 15% of pregnant women were aged between 18 and 49 (396/2,708); 18 pregnancies were documented for underage girls (15 to 17 years old, *n* = 125) and 370 pregnancies were documented for women aged 18 to 35 years. For 341 pregnancies age and origin (*n* = 2,675) were documented; six percent of them from Syria (164/2,675). Hyperemesis gravidarum (33/102) was the most frequent complication of pregnancy (ICD10 “O20-O29”), followed by pregnancy related pains (22/102) and bleeding (7/102) as well as dizziness/ weakness (6/102). Nine pregnancies were classified as high-risk pregnancies.

#### Psychological and behavioral disorders

The odds of being diagnosed with psychological and behavioral disorders (*n* = 454/13,902 all diagnoses) were generally three times higher in adults than in children, with age- and sex-specific data available [aOR 3.05 (95% CI: 2.22–4.168), *p* < 0.001]. The highest odds was in the 21–30 age group [36.2%, 154/425, aOR 3.91 (95% CI: 2.63–5.79), *p* < 0.001], followed by the 31–40 age group [23.10%, 98/125, aOR 3.74 (95% CI: 2.46–5.67), *p* < 0.001]. Consultations from male and female patients were equally affected. Among neurotic, stress-related and somatoform disorders (ICD10 “F40-F48”; *n* = 242) most important were post-traumatic stress disorder (PTSD), acute stress reactions (*n* = 138) and anxiety disorders (*n* = 41). Most relevant among children were PTSD and stress (*n* = 17), as well as disorders of psychological development (ICD10 “F80-F89”, *n* = 24), as autistic disorder (*n* = 9) and mixed specific developmental disorders (*n* = 6). For more details see Supplementary annex Table 8.

#### Dental problems

Dental problems were recorded in 2.7% of adult consultations (237/8,818 versus 1.5%, 64/4,317 of children; p < 0.001). Most frequent (80.4%, 242/301), in adults and children (80.2% and 54.7%), was toothache.

#### Other diseases

Most frequent (36%, 350/1,024 all diagnoses within “Other diseases”) in this superordinate category (1,024/13,902 all diagnoses) were consultations related to surgical or orthopedic follow-up care (see Supplementary annex Table 9). The odds of getting those diagnosis were four times higher for men [male: aOR 4.02 (95% CI: 2.86–5.64), adults: aOR 4.05 (95% CI: 2.70–6.06), *p* < 0.001].

Furthermore, 17.6% of consultations were for repeated prescriptions (ICD10 “Z76.0”, 180/1,023), e.g. long-term medication (83.3%, 150/180) or prescription of wheelchair, walking aid, prosthetic devices, and spectacles (*n* = 20).

No medical reason was documented for 208 consultations.

## Discussion

This study describes the spectrum of health problems, symptoms, and conditions of ASR who presented to three large outpatient clinics in Berlin from September 2015 to March 2016, a time with a high level of refugee movement in Europe. Today, 10 years later, Europe is once again confronted with a large number of refugees. The demand for medical care remains high, and sound decision-making for public health planning is of central importance. Therefore, we have decided to analyse this previously unevaluated dataset and to publish and discuss the results.

The outpatient clinics were set up by Charité University Hospital to treat acutely ill patients on the premises of two emergency refugee shelters and one refugee registration office. The strengths of those clinics lay in their quick availability immediately after a large number of ASR had arrived in Berlin and in their easy accessibility with free of charge on-site treatment five to six days a week. The limited resources, the need for a rapid response, the local conditions, and the patients' high mobility necessitated a syndromic approach. Extensive diagnostics were not feasible and might have delayed or even prevented treatment, as often only a single patient contact was possible.

We retrospectively analyzed data from 11,894 consultations (13,902 diagnoses) from September 2015 to March 2016. To our knowledge, there are just a few other studies with a similarly large scope, e.g. (18–20). A precise population denominator cannot be provided due to the high fluctuation and variability of duration of stay in the shelters, and because of a not quantifiable overlap between the populations attending the different clinics. Furthermore, it is important to acknowledge the possibility of multiple consultations per individual, as follow-up consultations were not consistently documented. Nevertheless, relative to the number of people whose arrival in Berlin was registered during the study period 43,000 the number of consultations is high. This indicates the high demand and the importance of low-threshold access to medical care for newly arriving ASR.

The study included a population comprising individuals originating from a diverse range of countries (*n* = 52). Consultations of young male patients between 21 and 40 years of age, mostly from Syria, Afghanistan, Iraq, Iran, formed the largest group. This reflects the demography of ASR arriving in Germany in 2015/2016 and Europe and coincides with reports from other studies (5, 6, 22, 23). In this context, although we are presenting a snapshot of the ASR situation in Germany in 2015/2016, the extent of the dataset and the representative, broad distribution of countries of origin indicate that the results can be carefully transferred to ASR arriving in other European countries, to similar situations or settings and at other times.

Most documented diseases corresponded to a ubiquitous general medical spectrum and could be treated on site in outpatient clinics. This has also been described in studies from other places in Germany (22, 24–30) and other European countries, such as Italy (31) and Malta (32). The observed disease spectrum comprised of many uncomplicated infections (e.g. non-specific respiratory tract infections or gastroenteritis) and common complaints (e.g. fever, pain), as well as serious and chronic conditions in less frequent numbers (e.g. heart disease or diabetes mellitus). In addition, pains, such as dorsalgia, limb pain and headaches, played a major role. Many consultations for pain were related to complaints concerning the musculoskeletal system and might have been caused by physical trauma and exhaustiveness of flight. The outpatient health service described above focused primarily on providing easy access for patients with acute complaints, which may explain why frequent infections, pain and orthopaedic problems were the most common diagnoses. The detection of chronic diseases and mental disorders could be addressed less in this setting.

The low number of hospital referrals shows that almost all diseases could be handled and treated by trained outpatient clinic staff, also observed in a similar setting in Germany (30). Alongside with the ubiquitous general medical spectrum a small number of rather rare and/or more complex diagnoses were recorded, such as malignancies and HIV infections. In addition, some conditions typical for the regions where most of the patients came from, such as sickle cell anemia, and thalassaemia were reported. The wide disease pattern underlines the interdisciplinary character of required refugee healthcare. Accordingly, a possibility of referral to specialized services for specific diagnosis and treatment of diseases beyond general medical spectrum need to be considered (e.g. gynecology and obstetrics, radiology, psychiatry, hematology) and be structurally feasible. The high number of pregnant women indicates a need for antenatal care.

There was no observable change in the frequency of communicable and non-communicable diseases over the course of the study period. Regarding infectious diseases, ASR are generally at risk of the same infectious diseases as the local population. However, due to the arduous journey, possible vaccinations gaps and the crowded conditions in shared accommodation, ASR are more susceptible to infectious diseases. The high proportion of mostly upper acute respiratory infections in the study population (36% of all diagnoses) corresponds to existing studies and reports (18, 19, 22, 24, 26, 33–35). The arrival of ASR in Berlin in autumn and winter and the corresponding weather conditions may have contributed to these findings, as well as the crowded housing conditions. Living in mass accommodations leads to high contact rates and thus to high transmission rates, especially for respiratory infections with high contagiosity (4, 36). Accordingly, Kühne et al. have shown that infectious disease outbreaks happen by far more frequently in the setting of mass accommodations than in the general population (36). The risk of transmission of infectious diseases in general or virus-related respiratory diseases is particularly high in the setting of reception centers and shared accommodation, as many people live together in a confined space and shared use of living, kitchen, dining, and sanitary facilities (37). Physical distancing measures cannot usually be implemented, or only to a limited extent (4, 38–40). Therefore, careful isolation of infectious patients, adequate sanitation and food and kitchen hygiene must be a priority. The stay in a mass accommodation, if not avoidable, must be kept as short as possible and vulnerable persons must be given priority for smaller accommodation units (39). Overall, adults sought medical help at similar frequencies for communicable (34%) and non-communicable diseases (27%). Expectedly, there were some differences among the disease profile between young and middle-aged versus older aged adults, particularly concerning non-communicable diseases such as CVD/CHD, COPD, and diabetes. Younger or middle-aged adults (age between 21 and 40 years) were more concerned by communicable diseases (37%) and older adults (age group 41 to >60) by non-communicable diseases, incl. orthopedic complaints, and pain (41%). This age-dependent distribution corresponds to the results from the German Health Update (GEDA 2019/2020-EHIS (41)), where prevalences of chronic diseases such as diabetes, coronary heart disease (CHD), stroke as well as COPD and osteoarthritis increase considerably from mid- to older adulthood. The most common reason for medical consultations among children were communicable diseases, accounting for 72% of consultations. RTI and diarrhea were most common, accounting for 18% of presentations in children under five years of age. This is not surprising as infectious diseases particularly acute RTI are among the most common infectious diseases in children (30) and respiratory and gastrointestinal infections have globally the highest incidence in children under 5 years of age (21, 42–44). In addition to age the prevalence of RTI in young ASR is also influenced by season and housing density (22, 45).

Considering that most patients came from high prevalence TB-countries, 42 TB cases seem not a large number. We hypothesise that the low number of presentations with TB was most likely due to the mandatory TB screening which was part of the initial physical examinations on arrival in accordance with §36(4) of the German Infection Protection Act (IfSG, 2001) (46). TB screening and follow-up were carried out by other service providers in consultations specifically targeting TB at a different location in Berlin. No data from these consultations is available in the scope of this study. Unfortunately, our data does not allow us to distinguish between patients who have previously been diagnosed with TB (either through screening or elsewhere), patients whose TB was missed by the TB screening and patients who had not yet undergone TB screening due to registration delays (7).

The low number of consultations for dental problems can be explained by the fact that no explicit dental care was offered and patients with tooth ache were referred elsewhere.

ASR are exposed to multiple and complex stressors, such as traumatic experiences in crisis areas and during flight, loss of relatives, and uncertain future prospects, that can have a lasting impact on their mental health. The risk of depression, anxiety and post-traumatic stress disorder (PTSD) is higher in ASR than in the host population. (47–50). In comparison with other reports (47, 51, 52) the proportion of psychological and behavioral disorders was relatively low in our study (3.3%). The focus of the clinics was clearly on acute somatic problems and diagnosing of mental disorders in this setting was challenging. Attending physicians had different levels of experience and training regarding mental health. A high workload, leading to a short consultation time per patient, facility structures not allowing sufficient privacy, and language barriers may have hindered psychological explorations and trustful conversations. We assume that some of the reported unspecific symptoms (gastrointestinal complaints, orthopedic pain or headaches) may indicate somatisation disorders due to mental stress (51, 53). Furthermore, most patients had only recently arrived, and mental health issues, particularly stress-related disorders, often become apparent after a longer period of residence. (53). Under the assumption that mental health problems among ASR were not adequately diagnosed and taken care of in the existing outpatient clinics, a central mental health refugee clinic came into operation in February 2016. Charité university hospital set up this clinic to better address language, resource, and knowledge barriers, as well as cultural aspects and stigmatization. Until summer of 2018, there were between 300 and 350 patient contacts per month (7). However, the findings reported elsewhere emphasize that the need for mental health support among ASR is high. It is likely that the need for mental health tends to be underestimated in acute outpatient clinics. Particularly in the context of psychosocial care for ASR, but also in medical care in general, the importance of language mediation for appropriate care has been emphasized (47, 54).

Regarding patient documentation, our study has shown that handwritten documentation may not be sufficient to adequately address medical documentation (including the coding of diagnoses (e.g. ICD10 codes) and monitoring of the health and care of ASR. Digitised patient records could also facilitate healthcare monitoring and follow-up in this setting. This need has also been identified in other studies in Germany, especially when patients move to other cities (55–57). The digitisation of patient records could facilitate the documentation and follow-up of illnesses that have already been diagnosed elsewhere, should ASR be required to move or be transferred to other federal states or facilities, e.g. Refugee Care Manage^®^ (RefCare^®^, project PRICARE) (55–57).

In accordance with current international standards, ASR should be offered healthcare services at the same level as the healthcare system in the host country (provided the countries fulfill the international standards of the WHO and the UNHCR (58)). This requires that ASR are entitled to receive care from the regular healthcare system (statutory health insurance) as early as possible after their arrival and that the healthcare system has the capacity to receive a large number of additional individuals (4, 40, 59, 60). The Berlin example shows that the local health system was not resilient enough in 2015/16 to meet the additional high demand for primary healthcare and mental health services in the face of a significant arrival of ASR. The Charité outpatient clinics played a key role in easing pressure on the healthcare system during this period by providing low-threshold primary healthcare with an essential filtering function. Shortly thereafter, in March 2016, the Arrival Center for Health Screening and Vaccination was established in Berlin, further strengthening the transition between initial care and the regular healthcare system—a role it continues to fulfill today. Before that, arriving ASR receive a mandatory initial medical examination, vaccination offer according to the recommendations by the German Standing Committee on Vaccination (STIKO), and medical care for as long as they are housed near the center. In Berlin, access to a health insurance card has also become available; in case of chronic conditions, enrolment in a health insurance scheme with access to standard healthcare can now mostly be arranged within days or weeks. The broadened examination, which includes non-communicable diseases, ensures that diagnoses and recommendations for further testing and treatment are effectively communicated to providers in the regular healthcare system. Each ASR receives a medical report for this purpose. Once insured, ASR are no longer treated within separate structures but access the same primary healthcare services as the general population. No additional dedicated medical resources are reserved for sudden increases in refugee numbers; instead, existing structures are scaled up as needed – a process that is now more manageable than it was prior to 2015/2016. (61) Nevertheless, providing healthcare for ASR in Germany remains challenging. For example, the electronic health card is not yet consistently implemented throughout the country. (62–64). Furthermore, a legislative amendment to the Asylum-Seekers Benefits Act (AsylbLG) in February 2024 means that ASR will receive only limited healthcare for a longer period than before (65–67). This has negative effects and leads, amongst other things, to lower uptake of healthcare services, increased misdiagnoses, missed opportunities for prevention, poorer long-term health outcomes for ASR and reduced chances of integration into society (64, 67).

### Strengths and limitations

The main strengths of this study lay in its large study population and high number of consultations resulting in a large data set. Another strong point is the source of the data: the data originate from unrestricted consultations in a low-threshold setting and were collected by healthcare professionals (doctors and midwives) for the purpose of acute medical care. This results in an unfiltered, authentic picture of the expressed medical needs.

Nevertheless, the study faces some limitations. Due to the high number of patients, the fact that each consultation was documented on a separate form, staff turnover in the outpatient clinics, and the technical setup (handwritten documentation forms were used at all sites; no software was employed), follow-up visits and repeat consultations could not always be reliably or consistently documented. We therefore assume that the number of documented follow-up consultations and repeat visits is underestimated. Therefore, it is not feasible to provide an accurate account of the number of patients treated and our findings refer to consultations or diagnoses and not to incidence/prevalence at the individual level. The denominator for setting the number of consultations in relation to the overall population is unclear. The number of ASR who arrived in Berlin over the study period can only approximate this denominator. This descriptive study has an exploratory focus and was not designed to detect any causal relations or predictions/ inferences. We cannot rule out residual confounding due to a lack of specific data on contextual factors like the time since arrival. Future research should aim to include these longitudinal and contextual parameters to better understand the observed effects. As some countries of origin were represented by very small numbers, we have grouped them together for clearer presentation. However, we are aware that this does not take into account the diversity within the regions, which could distort the comparison. In outpatient clinics with limited diagnostic capacities (see methods) there might occur a certain number of misdiagnoses. This limitation was reduced by ensuring that diagnoses were made by medical doctors or midwives with appropriate training and experience. For the study the ICD10-coding was performed retrospectively by the authors. The authors subjectively selected the level of detail of the ICD10 codes and assigned the syndromic diagnoses to ICD10 codes. To minimize miscoding, the authors discussed ambiguous and hard-to-read forms individually.

## Conclusion and recommendations

A large number of newly arrived ASR goes hand in hand with a high demand for medical care. The capacity of the local health system in 2015/16 was insufficient to address those additional needs, and access is difficult for many reasons. Even though our findings are based on data from 2015/16 and only reflect a proportion of demand within this population, representing those ASR who actually seek care, we conclude that providing quick, easily accessible healthcare close to the ASR' accommodation is particularly important. Most diseases in this population presented in this study correspond to a ubiquitous general medical spectrum and can be treated in relatively simply equipped outpatient clinics by physicians with a general medicine training. A timely and sufficient supply of primary healthcare is more important than the availability of specialists, as in such situations most patients seek treatment for minor illnesses and do not have access to the regular healthcare system. According to our findings, referrals to specialists and hospital care are rarely needed, but access must be ensured if they are indicated, especially for infants, older adults, and for antenatal care. To be resilient, a healthcare system requires not only specialists and hospitals, but also the capacity to rapidly scale up low-threshold primary healthcare on site, with the goal of preventing the existing healthcare system from becoming overburdened by the treatment of simple illnesses. This must be considered when preparing for future crisis responses, bearing in mind the circumstances faced by ASR. However, the above only refers to somatic medicine. The needs for mental healthcare might be high in ASR and cannot adequately be addressed in primary healthcare clinics. Facilities with a culturally sensitive approach, easy access and the availability of language mediation are essential, particularly for addressing mental health needs. Since the study was conducted, the technical requirements for language mediation have improved significantly and started to find their way into clinical practice. Overall, it is essential to strengthen cultural and migration-related competencies both within the healthcare sector and across all other areas of the public sector in order to foster the integration of different population groups. Only then can people receive appropriate care within the regular healthcare system.

## Data Availability

The raw data supporting the conclusions of this article will be made available by the authors, without undue reservation.

## Footnotes

1 Only in 2016. The numbers for the Republic of Moldova are not representative for Germany, as the registration of persons from the Republic of Moldova was guaranteed nationwide by the federal state of Berlin.
