# Supplementary material for: Disease spectrum and medical complaints among migrants: a study from three outpatient clinics for asylum seekers in Berlin, Germany
Source: Front Public Health. 2026 Jun 12;14:1774309. doi: 10.3389/fpubh.2026.1774309 (PMC13303496; doi:10.3389/fpubh.2026.1774309)
Supplement: Supplementary file 1 [file Supplementary_file_1.docx]

**Supplementary ANNEX**


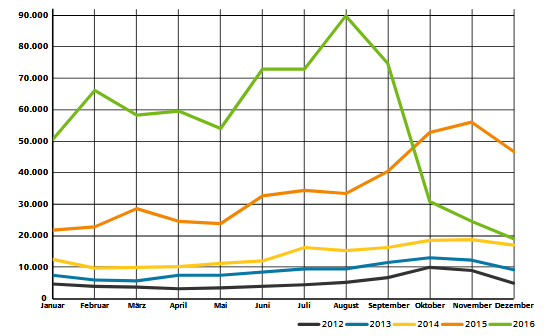


*Figure 1* (supplementary annex) *Development of the number of asylum applications year-on-year from 2012 to 2016 in Germany (numbers in persons)* Figure source: German Federal Office for Migration and Refugees: “Das Bundesamt in Zahlen 2016”, 2017, page 14 (Available online at: <https://www.bamf.de/SharedDocs/Anlagen/DE/Statistik/BundesamtinZahlen/bundesamt-in-zahlen-2016.pdf?__blob=publicationFile&v=16>; last access: 09.02.2024) (5)

| **Administrative data** |
| --- |
| Outpatient clinic registration number |
| “Green card” yes/no  ("Green Card": health insurance voucher for refugees that gives them access to health insurance cover; received after registration (2015)) |
| **Sociodemographic** **data** |
| Family name, name, date of birth/ age or adult/child, sex, nationality, language |
| Date and time of consultation |
| Location of consultation (outpatient clinics A-C) |
| Name of attending physician (Charité or voluntary personnel) |
| **Medical data** |
| (Leading) symptom |
| Medical history, emergency findings, self-medication |
| Pain scale |
| Allergies |
| Pregnancy (week of pregnancy, if applicable) |
| Vital signs: pulse, temperature, blood pressure, respiratory frequency, oxygen saturation, blood glucose level |
| body sizes: height/weight |
| On site diagnostic results: electrocardiogram (ECG), urine stix (leucocytes, glucose, erythrocytes, nitrite, pH value, protein), rapid test for StrepA and RSV |
| Brief findings/ course, diagnosis, ICD |
| Therapy/ medication administered |
| (Emergency) hospitalization |
| Follow-up consultation |
| Referral |

Table 1 (supplementary annex) Included variables of the documentation form (handwritten) used in three refugee outpatient clinics in Berlin, 2015/2016

| **Chi^2^-test** |
| --- |
| - *tab [var1] [var2], chi2* |
| **Logistic regression** |
| - *“logistic [diagnosis] i.sex i.agegroup i.origin_nomis2 ib[#largest population group].origin_nomis2”* |
| - *“logistic [diagnosis] i.sex i.adult_child i.origin_nomis2 ib[#largest population group].origin_nomis2”* |
| - *“logistic [diagnosis] i.sex ib1.sex i.adult_child i.origin_nomis ib[#largest population group].origin_nomis2”* |

Table 2 (supplementary annex) Descriptive and analytic data analysis

| **Age of ASR in age groups** | **Number** | **Percent** |
| --- | --- | --- |
| 0 to 10 years | 3,085 | 27.5 |
| 11 to 20 years | 1,449 | 12.9 |
| 21 to 30 years | 2,925 | 26.0 |
| 31 to 40 years | 1,861 | 16.6 |
| 41 to 50 years | 997 | 8.9 |
| 51 to 60 years | 623 | 5.5 |
| > 60 years | 293 | 2.6 |
| **Total*** | **11,233** | **100** |

* Total number of consulting ASR for which data on sex was available.

Table 3 (supplementary annex) Age of consulting ASR in age groups (available information for n = 11,233 consultations) at three refugee outpatient clinics in Berlin, 2015/2016

| **Number** | **Country of origin** | **Group** | **Frequency** |
| --- | --- | --- | --- |
| 1 | Syria | - | 4,263 |
| 2 | Afghanistan | - | 2,261 |
| 3 | Iraq | - | 1,576 |
| 4 | Iran | - | 489 |
| 5 | Moldova | Balkans | 269 |
| 6 | Pakistan | - | 197 |
| 7 | Serbia | Balkans | 114 |
| 8 | Lebanon | Other | 99 |
| 9 | Palestine | Other | 97 |
| 10 | Egypt | North Africa | 85 |
| 11 | Albania | Balkans | 79 |
| 12 | Turkmenistan | CIS (former Soviet republics in Eurasia) | 69 |
| 13 | Bosnia | Balkans | 62 |
| 14 | Libya | North Africa | 43 |
| 15 | Somalia | East Africa | 40 |
| 16 | Russia | CIS (former Soviet republics in Eurasia) | 38 |
| 17 | Eritrea | East Africa | 32 |
| 18 | Turkey | Other | 31 |
| 19 | Kosovo | Balkans | 27 |
| 20 | Ukraine | CIS (former Soviet republics in Eurasia) | 25 |
| 21 | Vietnam | Other | 12 |
| 22 | Chechnya | CIS (former Soviet republics in Eurasia) | 11 |
| 23 | Senegal | West Africa | 8 |
| 24 | Algeria | North Africa | 7 |
| 25 | Armenia | CIS | 7 |
| 26 | The Gambia | West Africa | 7 |
| 27 | Morocco | North Africa | 7 |
| 28 | Kenya | East Africa | 6 |
| 29 | Nigeria | West Africa | 6 |
| 30 | Romania | Balkans | 6 |
| 31 | Tunisia | North Africa | 6 |
| 32 | Belarus | CIS (former Soviet republics in Eurasia) | 4 |
| 33 | Macedonia | Balkans | 4 |
| 34 | Bangladesh | Other | 3 |
| 35 | Georgia | CIS (former Soviet republics in Eurasia) | 3 |
| 36 | Yemen | Other | 3 |
| 37 | Cameroon | Central Africa | 3 |
| 38 | Azerbaijan | CIS (former Soviet republics in Eurasia) | 2 |
| 39 | Bulgaria | Balkans | 2 |
| 40 | Equatorial Guinea | Central Africa | 2 |
| 41 | Guinea-Bissau | West Africa | 2 |
| 42 | Sudan | East Africa | 2 |
| 43 | Djibouti | East Africa | 1 |
| 44 | Ethiopia | East Africa | 1 |
| 45 | Ghana | West Africa | 1 |
| 46 | Kazakhstan | CIS (former Soviet republics in Eurasia) | 1 |
| 47 | Kurdistan | Other | 1 |
| 48 | Latvia | Other | 1 |
| 49 | Nepal | Other | 1 |
| 50 | Tajikistan | CIS (former Soviet republics in Eurasia) | 1 |
| 51 | Uganda | East Africa | 1 |
| 52 | Uzbekistan | CIS (former Soviet republics in Eurasia) | 1 |
| **Total number of countries of origin** | | | **10,019** |

Table 4 (supplementary annex) Country of origin, grouping and frequency of patients (available information for
n = 10,019 consultations) at three refugee outpatient clinics in Berlin, 2015/2016

| **Superordinate categories** | | | | **≥18 years** | | **< 18 years** | | **Total*** | |
| --- | --- | --- | --- | --- | --- | --- | --- | --- | --- |
|  |  |  |  | **n** | **%** | **n** | **%** | **n** | **%** |
| 1 | | Infectious diseases | | 3,012 | 34.7 | 3,129 | 72.5 | **6,502** | **46.8** |
| 2 | | Non-infectious internal medical diseases | | 2,384 | 27.1 | 574 | 13,3 | **3,128** | **22.5** |
| 3 | | Orthopaedic conditions | | 820 | 9.3 | 114 | 2.6 | **980** | **7.1** |
| 4 | | Pains | | 778 | 8.8 | 64 | 1.5 | **893** | **6.4** |
| 5 | | Gynaecological conditions (non-infectious) & childbirth | | 523 | 5.8 | 26 | 0.6 | **595** | **4.3** |
| 6 | | Psychological and behavioural disorders | | 372 | 4.4 | 53 | 1.3 | **454** | **3.3** |
| 7 | | Dental problems | | 237 | 2.7 | 64 | 1.5 | **326** | **2.3** |
| 8 | | Other | | 692 | 7.9 | 293 | 6.8 | **1,024** | **7.4** |
| **Total** | | | | **8,818** | **100** | **4,317** | **100** | **13,902** | **100** |
| **ICD10 codes per chapter of classification of the ICD** | | | | **≥18 years** | | **< 18 years** | | **Total*** | |
| **No.** | **ICD10** | | **Name** | **n** | **%** | **n** | **%** | **n** | **%** |
| I | A00-B99 | | Certain infectious and parasitic diseases | 483 | 5.3 | 686 | 15.6 | **1,236** | 8.8 |
| II | C00-D49 | | Neoplasms | 110 | 1.2 | 5 | 0.1 | **117** | 0.8 |
| III | D50-D89 | | Diseases of the blood and blood-forming organs and certain disorders involving the immune mechanism | 40 | 0.5 | 22 | 0.6 | **63** | 0.5 |
| IV | E00-E89 | | Endocrine, nutritional and metabolic diseases | 233 | 2,5 | 25 | 0.5 | **268** | 1.9 |
| V | F01-F99 | | Mental, behavioral and neuro-developmental disorders | 380 | 4,5 | 57 | 1.4 | **466** | 3.4 |
| VI | G00-G99 | | Diseases of the nervous system | 381 | 4,2 | 70 | 1.6 | **481** | 3.5 |
| VII | H00-H59 | | Diseases of the eye and adnexa | 142 | 1.6 | 75 | 1.7 | **228** | 1.6 |
| VIII | H60-H95 | | Diseases of the ear and mastoid process | 101 | 1.1 | 121 | 2.9 | **231** | 1.7 |
| IX | I00-I99 | | Diseases of the circulatory system | 440 | 5.0 | 37 | 0.9 | **497** | 3.6 |
| X | J00-J99 | | Diseases of the respiratory system | 2,354 | 26.7 | 2,272 | 52.5 | **4,888** | 35.1 |
| XI | K00-K95 | | Diseases of the digestive system | 723 | 8.2 | 117 | 2.7 | **898** | 6.5 |
| XII | L00-L99 | | Diseases of the skin and subcutaneous tissue | 335 | 4.0 | 187 | 4.4 | **575** | 4.2 |
| XIII | M00-M99 | | Diseases of the musculoskeletal system and connective tissue | 929 | 10.7 | 58 | 1.3 | **1,025** | 7.4 |
| XIV | N00-N99 | | Diseases of the genitourinary system | 404 | 4.6 | 56 | 1.3 | **488** | 3.5 |
| XV | O00-O9A | | Pregnancy, childbirth and the puerperium | 112 | 1.3 | 5 | 0.1 | **125** | 0.9 |
| XVI | P00-P96 | | Certain conditions originating in the perinatal period | - | - | 2 | 0.05 | **2** | 0.01 |
| XVII | Q00-Q99 | | Congenital malformations, deformations and chromosomal abnormalities | 3 | 0.03 | 34 | 0.8 | **37** | 0.3 |
| XVIII | R00-R99 | | Symptoms, signs and abnormal clinical and laboratory findings, not elsewhere classified | 363 | 4.1 | 217 | 5.2 | **624** | 4.5 |
| XIX | S00-T88 | | Injury, poisoning and certain other consequences of external causes | 371 | 4.2 | 105 | 2.4 | **510** | 3.7 |
| XX | V00-Y99 | | External causes of morbidity | 30 | 0.3 | 3 | 0.07 | **35** | 0.2 |
| XXI | Z00-Z99 | | Factors influencing health status and contact with health services | 882 | 10.0 | 167 | 3.8 | **1,106** | 8.0 |
| XXII |  | | Codes for special purposes | 2 | 0.02 | - | - | **2** | 0.01 |
| **Total** **of diagnoses** **in disease categories and ICD10 codes** | | | | **8,818** | **100** | **4,317** | **100** | **13,902** | **100** |

*The total comprises of the sum of the two first columns plus the diagnoses for which age was not indicated.

Table 5 (supplementary annex) Distribution of diagnoses (n = 13,902) in disease categories and ICD10 codes by age groups among refugees consulting at three outpatient clinics in Berlin, 2015/2016

| **Disease group – Orthopaedics** | | **≥18 years** | | **< 18 years** | | **Total all*** | |
| --- | --- | --- | --- | --- | --- | --- | --- |
|  |  | **n** | **%** | **n** | **%** | **n** | **%** |
| **Disorders musculoskeletal system, soft tissue, spine/back** | | **283** | **17.7** | **20** | **11.2** | **309** | **16.5** |
| M40-M53** | Dorsopathies (excl. back pain/ lumbago) | 150 | 18.3 | 2 | 1.7 | 154 | 15.7 |
| M60-M79.9*** | Soft tissue disorders | 127 | 15.5 | 18 | 15.8 | 149 | 15.2 |
| M95-M99 | Other disorders of the musculoskeletal system and connective tissue | 6 | 0.7 | - | - | 6 | 0.6 |
| **Injuries** | | **236** | **14.8** | **56** | **31.5** | **318** | **17.1** |
| S40-S99 | Injuries to the shoulder and upper arm/elbow and forearm/wrist and hand/hip and thigh/knee and lower leg/ankle and foot | 154 | 18.8 | 28 | 24.6 | 194 | 19.8 |
| T08-T14 | Injuries to unspecified part of trunk, limb or body region | 55 | 6.7 | 21 | 18.4 | 86 | 8.8 |
| S00-S39 | Injuries to the head/neck/thorax/abdomen, lower back, lumbar spine and pelvis | 23 | 2.8 | 7 | 6.1 | 33 | 3.4 |
| T00-T07 | Injuries involving multiple body regions | 4 | 0.5 | - | - | 5 | 0.5 |
| **Disorders joints/cartilage** | | **182** | **11.4** | **7** | **3.93** | **196** | **10.5** |
| M00-M25 | Arthropathies | 156 | 19.0 | 5 | 4.4 | 168 | 17.1 |
| M80-M94**** | Osteopathies and chondropathies | 16 | 1.9 | 1 | 0.1 | 17 | 1.7 |
| M86 | Osteomyelitis | 10 | 1.2 | 1 | 0.1 | 11 | 1.1 |
| **Fractures** | | **73** | **4.6** | **14** | **7.9** | **89** | **4.7** |
| T10-T12 | Upper/ lower limb level unspecified/ Other injuries of upper limb level unspecified | 58 | 7.1 | 12 | 10.5 | **72** | **7.3** |
| S02,S12,S22,S32 | skull and facial bones/neck/rib(s), sternum, thoracic spine/lumbar spine, pelvis | 14 | 1.7 | 2 | 1.7 | **16** | **1.6** |
| T02 | involving multiple body regions | 1 | 0.1 | - | - | **1** | **0.1** |
| **Other** | | **46** | **2.9** | **17** | **9.5** | **68** | **3.6** |
| Y36.-! | War operations, unspecified | 30 | 3.7 | 2 | 1.7 | **34** | **3.5** |
| T20-T32 | Burns and corrosions | 8 | 1.0 | 10 | 8.8 | **20** | **2.0** |
| S06.0 | Concussion | 3 | 0.4 | 2 | 1.7 | **6** | **0.6** |
| T66-T78 | Other and unspecified effects of external causes | 3 | 0.4 | 3 | 2.6 | **6** | **0.6** |
| T15-T19 | Effects of foreign body entering through natural orifice | 2 | 0.2 | - | - | **2** | **0.2** |
| **Total** **of ICD10 codes of subcategory “Orthopaedics”** | | **820** | **100** | **114** | **100** | **980** | **100** |

* The total comprises of the sum of the two first columns plus the diagnoses for which age was not indicated.

** ICD10 group modified by author (original ICD10 “M40-M54”), “M54,-“subsumed among disease group pain separately.
*** ICD10 group modified by author (original ICD10 “M60-M79”), i. e. exclusive “M79.6”, “M79.6,
 subsumed among disease group “pain” separately.
**** ICD10 group modified by author (original ICD10 “M80-M94”), i. e. exclusive “M86”, “M86”, subsumed among
 disease group “orthopaedics” separately.

Table 6 (supplementary annex) List of ICD10 codes of subcategory “Orthopaedics” in total and by age (n = 980 diagnoses) among refugees consulting at three outpatient clinics in Berlin, 2015/2016

| **ICD10 code** | **Title ICD10 code** | **≥18 years** | | **< 18 years** | | **Total all*** | |  |
| --- | --- | --- | --- | --- | --- | --- | --- | --- |
|  |  | **n** | **%** | **n** | **%** | **n** | **%** |  |
| **Z34** | **Supervision of normal pregnancy** | **285** | **54.5** | **13** | **50.0** | **323** | **54.3** |  |
| N80-N98 | Non-inflammatory disorders of female genital tract | 99 | 18.9 | 8 | 30.8 | 113 | 19.0 |  |
| O20-O29 | Other maternal disorders predominantly related to pregnancy | 93 | 17.8 | 4 | 15.4 | 102 | 17.1 |  |
| Z30-Z39 | Persons encountering health services in circumstances related to reproduction | 11 | 2.1 | - | - | 13 | 2.2 |  |
| O85-O92 | Complications predominantly related to the puerperium | 7 | 1.3 | - | - | 10 | 1.7 |  |
| N70-N77 | Inflammatory diseases of female pelvic organs | 14 | 2.7 | - | - | 18 | 3.0 |  |
| O00-O08 | Pregnancy with abortive outcome | 6 | 1.2 | - | - | 6 | 1.0 |  |
| O30-O48 | Maternal care related to the fetus and amniotic cavity and possible delivery problems | 5 | 1.0 | 1 | 3.8 | 6 | 1.0 |  |
| Z33! | Pregnant state, incidental | 2 | 0.4 | - | - | 3 | 0.5 |  |
| O10-O16 | Oedema, proteinuria and hypertensive disorders in pregnancy, childbirth and the puerperium | 1 | 0.2 | - | - | 1 | 0.2 |  |
| **Total of ICD10 codes of subcategory “Gynecology (non-communicable) and obstetrics”** | | **523** | **100** | **26** | **100** | **595** | **100** |  |

*The total comprises of the sum of the two first columns plus the diagnoses for which age was not indicated.

Table 7 (supplementary annex) List of ICD10 codes of subcategory “Gynecology (non-communicable) and obstetrics” in total and by age (n = 595 diagnoses) among refugees consulting at three outpatient clinics in Berlin, 2015/2016

| **ICD10 code** | **Title ICD10 code** | **≥18 years** | | **< 18 years** | | **Total all*** | |  |
| --- | --- | --- | --- | --- | --- | --- | --- | --- |
|  |  | **n** | **%** | **n** | **%** | **n** | **%** |  |
| **F40-F48** | **Neurotic, stress-related and somatoform disorders (incl. PTSD)** | **215** | **57.8** | **22** | **44.6** | **242** | **53.3** |  |
| F30-F39 | Mood (affective) disorders | 66 | 17.7 | 1 | 1.8 | 75 | 16.4 |  |
| F80-F89 | Disorders of psychological development | 7 | 1.9 | 24 | 42.9 | 32 | 7.0 |  |
| F10-F19 | Mental and behavioural disorders due to psychoactive substance use | 24 | 6.5 | - | - | 29 | 6.4 |  |
| F50-F59 | Behavioural syndromes associated with physiological disturbances and physical factors | 22 | 5.9 | 1 | 1.8 | 28 | 6.1 |  |
| F99 | Unspecified mental disorder | 17 | 4.6 | 3 | 5.4 | 24 | 5.3 |  |
| F20-F29 | Schizophrenia, schizotypal and delusional disorders | 12 | 3.2 | - | - | 13 | 2.8 |  |
| F03 | Unspecified dementia | 4 | 1.1 | - | - | 4 | 0,9 |  |
| F60-F69 | Disorders of adult personality and behaviour | 2 | 0.5 | 1 | 1.8 | 3 | 0.7 |  |
| F00-F09 | Organic, including symptomatic, mental disorders | 2 | 0.5 | - | - | 2 | 0.4 |  |
| F90-F98 | Behavioural and emotional disorders with onset usually occurring in childhood and adolescence | 1 | 0.3 | 1 | 1.8 | 2 | 0.4 |  |
| **Total of ICD10 codes of subcategory “Psychological and behavioural disorders”** | | **372** | **100** | **53** | **100** | **454** | **100** |  |

*The total comprises of the sum of the two first columns plus the diagnoses for which age was not indicated.

Table 8 (supplementary annex) List of ICD10 codes of subcategory “Psychological and behavioural disorders” in total and by age (n = 454 diagnoses) among refugees consulting at three outpatient clinics in Berlin, 2015/2016

| **ICD10 code** | **Title ICD10 code** | **≥18 years** | | **< 18 years** | | **Total all*** | |
| --- | --- | --- | --- | --- | --- | --- | --- |
|  |  | **n** | **%** | **n** | **%** | **n** | **%** |
| **Z01** | **Other special examinations/ investigations of persons without complaint or reported diagnosis** | **123** | **18.0** | **91** | **36.3** | **219** | **21.4** |
| Z76.0 | Issue of repeat prescription | 149 | 21.8 | 21 | 8.4 | **180** | **17.6** |
| Z48.9 | Surgical follow-up care, unspecified | 142 | 20.8 | 9 | 3.6 | **156** | **15.2** |
| Z48.0 | Attention to surgical dressings and sutures | 116 | 17.0 | 17 | 6.8 | **141** | **13.8** |
| R40-R46 | Symptoms and signs involving cognition, perception, emotional state and behaviour | 51 | 6.7 | 5 | 2.4 | **60** | **5.9** |
| R50.9 | Fever, unspecified | 4 |  | 43 |  | **51** | **5.0** |
| Z09.4 | Follow-up examination after treatment of fracture | 41 | 6.0 | 5 | 2.0 | **46** | **4.5** |
| Q00-Q99 | Congenital malformations, deformations and chromosomal abnormalities | 3 | 0.4 | 34 | 13.5 | **37** | **3.6** |
| R63.- | Symptoms and signs concerning food and fluid intake | 2 | 0.3 | 25 | 10.0 | **27** | **2.6** |
| T88.1 | Other complications following immunization, not elsewhere classified | 12 | 1.8 | 14 | 5.2 | **26** | **2.5** |
| F70-F79 | Mental retardation | 8 | 1.2 | 4 | 1.6 | **12** | **1.2** |
| T14.03 | Superficial injury of unspecified body region (incl. insect bite) | 8 | 1.2 | 4 | 1.6 | **12** | **1.2** |
| R25-R29 | Symptoms and signs involving the nervous and musculoskeletal systems | 10 | 1.5 | - | - | **10** | **1.0** |
| Z20-Z29 | Persons with potential health hazards related to communicable diseases | 3 | 0.4 | 4 | 1.6 | **7** | **0.7** |
| Z47.- | Other orthopaedic follow-up care | 1 | 0.1 | 6 | 2.4 | **7** | **0.7** |
| R60.9 | Oedema, unspecified | 4 | 0.6 | - | - | **5** | **0.5** |
| Z70-Z76 | Persons encountering health services in other circumstances | 3 | 0.4 | 1 | 0.4 | **5** | **0.5** |
| R62.- | Lack of expected normal physiological development | - | - | 4 | 1.6 | **4** | **0.4** |
| P00-P96 | Certain conditions originating in the perinatal period | - | - | 2 | 0.8 | **2** | **0.2** |
| R59.- | Enlarged lymph nodes | 1 | 0.1 | 1 | 0.4 | **2** | **0.2** |
| T36-T50 | Poisoning by drugs, medicaments and biological substances | 2 | 0.3 | - | - | **2** | **0.2** |
| T88.7 | Unspecified adverse effect of drug or medicament | 1 | 0.1 | 1 | 0.4 | **2** | **0.2** |
| Z80-Z99 | Persons with potential health hazards related to family/ personal history, certain conditions influencing health status | 2 | 0.3 | - | - | **2** | **0.2** |
| Z93.- | Artificial opening status | 2 | 0.3 | - | - | **2** | **0.2** |
| U80-U85 | Resistance to antimicrobial and antineoplastic drugs | 2 | 0.3 | - | - | **2** | **0.2** |
| G70-G73 | Diseases of myoneural junction and muscle | - | - | 1 | 0.4 | **1** | **0.1** |
| R61.- | Hyperhidrosis | 1 | 0.1 | - | - | **1** | **0.1** |
| V01-X59 | Accidents | - | - | 1 | 0.4 | **1** | **0.1** |
| Z55-Z65 | Persons with potential health hazards related to socioeconomic and psychosocial circumstances | 1 | 0.1 | - | - | **1** | **0.1** |
| **Total** **of ICD10 codes of subcategory “Other diseases”** | | **692** | **100** | **293** | **100** | **1,024** | **100** |

*The total comprises of the sum of the two first columns plus the diagnoses for which age was not indicated.

*Table 9 List of ICD10 codes of subcategory “Other diseases” in total and by age (n = 1,023 diagnoses) among refugees consulting at three outpatient clinics in Berlin, 2015/2016*
